# The Development of Silver Nanoparticles-Based Colorimetric Uric Acid Detection: An Extension Study of Silver Nanoparticles Extraction from Water Hyacinth (*Eichhornia crassipes*)

**DOI:** 10.3390/bios16080409

**Published:** 2026-07-29

**Authors:** Fueangfakan Chutrakulwong, Mana Intarasawang, Kheamrutai Thamaphat

**Affiliations:** 1 Division of Physics, Faculty of Science and Technology, Rajamangala University of Technology Krungthep, Bangkok 10120, Thailand; fueangfakan.c@mail.rmutk.ac.th; 2 Department of Science and Technology, Suksanari School, Bangkok 10600, Thailand; mana_in@snr.ac.th; 3 iNest Research and Innovation Center, Supavut Industry Company Limited, Chon Buri 20150, Thailand

**Keywords:** silver nanoparticles, colorimetric biosensor, uric acid detection, uricase-based sensing, green synthesis, water hyacinth extract, plasmonic sensing

## Abstract

This work is an extension of our previous work on UV-assisted green synthesis of silver nanoparticles (AgNPs) from water hyacinth leaf extract, which is focused on using their unique biogenic capping layer to mitigate matrix interference in clinical diagnostics. The synthesized AgNPs were evaluated for uric acid (UA) detection and used to fabricate a plasmonic colorimetric biosensing platform. The results show that the biosynthesized AgNPs effectively act as optical signal transducers in an uricase-based enzymatic system, in which the natural capping shield provides excellent electrosteric protection, providing excellent colloidal stability without non-specific aggregation in complex matrices. A linear correlation between absorbance and UA concentration was observed in the range of 100–500 µM (R^2^ = 0.99). The limit of detection (LOD) calculated by the 3σ/slope approach was 25.89 µM. The sensor showed good repeatability with a relative standard deviation (RSD) below 5%, and stability studies showed that the AgNPs retained more than 90% of their initial response after 14 days. Recovery studies in spiked human serum showed satisfactory accuracy (96.3–103.4%), confirming a high tolerance to endogenous interfering species (e.g., ascorbic acid and glutathione) without pre-purification steps. Importantly, the working range covers clinically relevant uric acid concentrations found in human serum. The obtained results point to the potential of waste-derived green-stabilized AgNPs as a robust plasmonic-based colorimetric biosensing platform for the clinical monitoring of uric acid, successfully combining ecological valorization with high-performance, matrix-tolerant diagnostics.

## 1. Introduction

Purines are indispensable biomolecules that perform a wide range of critical functions within the cell, with their most prominent role being the formation of the monomeric building blocks of nucleic acids, DNA and RNA. Beyond their genetic significance, purine derivatives are actively involved in regulating cellular energy metabolism and signal transduction pathways, and they constitute essential structural components of several coenzymes. Purines have also been shown to play important physiological roles in diverse tissues, including the regulation of platelet function, muscle activity, and neurotransmission. To maintain normal growth, proliferation, and survival for cells, a tightly regulated balance of purine availability is required [1,2,3]. Under physiological conditions, this balance is achieved through the coordinated action of enzymes that monitor purine metabolism, which precisely regulate the rates of purine synthesis and degradation. Purine nucleotides generated through biosynthetic pathways are ultimately directed into catabolic pathways, where they undergo sequential enzymatic degradation to form uric acid (UA), the terminal product of purine nucleotide metabolism [4,5].

The blood normally breaks down UA, which is then excreted as urine by the kidneys [6]. Generally, men have serum uric acid levels of 150–420 µM and women have levels of 90–360 µM [7]. However, serum UA levels may increase if the body produces too much UA or if the body cannot remove enough UA from the body quickly. This disorder is called hyperuricemia [8,9,10] and has been associated with various illnesses and early death [11,12]. Symptoms of hyperuricemia include kidney stones, tophi and gout [8,13]. Additionally, heart failure [14,15,16,17], coronary artery disease, stroke [18,19,20], and hypertension have all been linked to asymptomatic hyperuricemia. Furthermore, it has been linked to chronic kidney disease (CKD), acute kidney injury (AKI) [21,22], and a more notable decrease in the global filtration rate in preclinical models [19]. Therefore, researchers working in the field of healthcare and disease diagnosis have been particularly interested in the identification of UA [23].

In the early stages, the phosphotungstic acid method was extensively employed for UA determination [24]. Later on, a variety of detection strategies have been developed with the advancement of analytical techniques and increased insight into UA-related chemistry, including electrochemical sensing [25], chromatographic techniques [26], capillary electrophoresis [27], fluorescence-based assays [28], paper-based analytical platforms [29], colorimetric approaches [30,31,32,33], and molecular spectroscopy [34]. Despite their high analytical performance, electrochemical and chromatographic methods are often constrained by complex instrumentation, high operational costs, time-intensive procedures, and susceptibility to interference, thereby limiting their suitability for rapid, routine analysis [25,26]. In contrast, colorimetric detection has garnered substantial attention owing to its simple visual readout, rapid response, operational convenience, high sensitivity, and low reagent consumption [34,35]. Consequently, colorimetric methods have emerged as a powerful and versatile analytical tool and are now widely applied across environmental monitoring, biological analysis, and clinical diagnostics.

In recent years, nanoparticle-based colorimetric biosensors employing metals, metal oxides, semiconductors, and hybrid nanomaterials have attracted considerable attention for applications in environmental monitoring, biological analysis, and clinical diagnostics. Parallel advances in the ligand chemistry of metal nanoparticles have enabled the rational design of highly selective and sensitive colorimetric sensing platforms capable of recognizing a wide range of chemical and biomolecular targets, often allowing for direct visual readout without the need for sophisticated instrumentation [36,37]. Among noble metal nanomaterials, silver nanoparticles (AgNPs) have emerged as particularly promising candidates for visual sensing applications [38,39,40]. This distinction arises from their exceptionally high extinction coefficients and the tunable nature of their localized surface plasmon resonance (LSPR), which can be precisely modulated by controlling particle size and interparticle spacing [41,42]. These advantageous plasmonic characteristics make AgNPs highly effective signal transducers in colorimetric sensors, producing distinct and easily discernible color changes observable by the naked eye [43].

The green synthesis of AgNPs has recently received much attention as an environmentally benign, economical and sustainable alternative to conventional physical and chemical reduction methods. A variety of plant extracts have been successfully used for the synthesis of AgNPs under ambient or thermal conditions without any light irradiation, using plants such as *Camellia sinensis* (green tea) [44], *Azadirachta indica* (neem) [45] and *Aloe vera* [46] which act as reducing and stabilizing agents simultaneously. The integration of ultraviolet (UV) irradiation is being actively explored by researchers to improve this biogenic process. UV-assisted green synthesis utilizes photochemical energies to significantly enhance the rate of reduction of silver ions (Ag^+^ to Ag^0^) mediated by plant phytoconstituents compared to conventional dark or ambient-light plant synthesis [47]. In addition to reducing the reaction time from hours to minutes, this photo-activation is vital for controlling the shape of the nanoparticles, generally producing more crystalline, smaller and more monodisperse AgNPs with improved LSPR properties [48].

Compared to the conventional chemically stabilized AgNPs, which generally require multi-step functionalization and suffer from stability problems in biological fluids, the multi-functional biomolecules present in *Eichhornia crassipes* extract can provide a dual advantage not only as a green reducing agent but also as a durable natural capping shield. This unique biogenic interface provides specific electrosteric protection to minimize matrix-induced non-specific aggregations in complex biological environments such as human serum.

In our previous investigation, entitled “Investigating UV-Irradiation Parameters in the Green Synthesis of Silver Nanoparticles from Water Hyacinth Leaf Extract: Optimization for Future Sensor Applications” [49], the water hyacinth leaf extract was systematically optimized by varying UV-irradiation conditions. Key reaction parameters, including irradiation time, solution pH, and the choice of stabilizing agent, were carefully examined. The results demonstrated that optimal AgNPs formation was achieved at a pH of 8.5 in the presence of starch as a stabilizer, with one hour of UV-A exposure. Under these conditions, AgNPs with the smallest particle size and highest yield were obtained. This study underscored the feasibility of employing water hyacinth as an environmentally benign, low-cost reducing agent for AgNP synthesis, highlighting its promising potential for applications in pharmaceutical formulations and sensing technologies [50,51]. The current work is an extension of our previous research, focusing on the potential of the synthesized AgNPs for UA detection and their use in developing a UA colorimetric detection, the details of which are as follows.

## 2. Materials and Methods

### 2.1. Materials

Fresh water hyacinth plants (*Eichhornia crassipes*) with clean and intact leaves were collected from a canal in Bangkok, Thailand. Silver nitrate (AgNO_3_, 99.9% purity), uric acid (≥99%, crystalline), and uricase (≥2 units/mg) were obtained from Sigma-Aldrich (St. Louis, MO, USA). Deionized (DI) water was supplied by RCI Labscan (Bangkok, Thailand), and Whatman No. 1 filter paper (GE Healthcare, Little Chalfont, UK) was used for filtration. Ultraviolet irradiation was conducted using a UV-A lamp (10 W, λ = 365 nm; Tokiwa, Tokyo, Japan). Soluble starch and disodium hydrogen orthophosphate (Na_2_HPO_4_·12H_2_O) were purchased from Ajax Finechem (Auckland, New Zealand). Sodium dihydrogen phosphate dihydrate (NaH_2_PO_4_·2H_2_O) was obtained from Quality Reagent Chemical (QRëC, Auckland, New Zealand). Peroxide test strips were obtained from Merck KGaA (Darmstadt, Germany). All glassware used in this study was heat-treated before the experiments.

### 2.2. Preparation of Substances

#### 2.2.1. Water Hyacinth Extraction

The water hyacinth leaves from a natural water source were washed and soaked in DI water at room temperature (25 °C) for 15 min. After that, they were air-dried at room temperature. Once the leaves were dry, they were cut into small pieces of approximately 1 × 1 cm. Then, 10 g of water hyacinth leaves and 100 mL of DI water were put in a 250 mL Erlenmeyer flask and heated at 100 °C for 30 min. The mixture was then filtered using Whatman No. 1 filter paper. The extract obtained from the water hyacinth leaves was subsequently used for the synthesis of AgNPs.

#### 2.2.2. AgNO_3_ Solution

AgNO_3_ solution with a concentration of 10 mM was prepared by dissolving 0.17 g of AgNO_3_ in 100 mL of DI water.

#### 2.2.3. Starch Solution

A starch solution was prepared to use as a stabilizing agent at a concentration of 1.5% (*w*/*v*) by dissolving 1.5 g of soluble starch in 100 mL of DI water. The solution was stirred thoroughly using a magnetic stirrer and then autoclaved to enhance starch dissolution.

#### 2.2.4. Buffer Solution

NaH_2_PO_4_·2H_2_O (2.76 g) was dissolved in 1000 mL of deionized water. Na_2_HPO_4_·12H_2_O was separately dissolved in deionized water (1000 mL). NaH_2_PO_4_·2H_2_O and Na_2_HPO_4_·12H_2_O were mixed in a 19:81 (*v*/*v*) ratio to obtain a buffer solution at pH 7.4. The remaining stock solutions were stored at 2–8 °C.

#### 2.2.5. Uric Acid Solution

Monosodium urate (MSU) crystals (0.042 g) were weighed and dissolved in 50 mL of phosphate buffer solution (pH 7.4). The solution was stirred with a magnetic stirrer and heated to 90 °C until the MSU crystals were completely dissolved, yielding a uric acid solution with a concentration of 5 mM. The prepared solution was stored in a refrigerator for subsequent experiments.

#### 2.2.6. Uricase Solution

Uricase powder (0.0013 g) was weighed and dissolved in 10 mL of phosphate buffer (pH 7.4). The prepared solution was stored at −4 °C for subsequent experiments.

#### 2.2.7. Determination of Hydrogen Peroxide

A 5 mM uric acid solution was diluted with phosphate buffer solution (pH 7.4) to obtain final concentrations of 100, 200, 300, 400, and 500 µM. For each concentration, 6 mL of the uric acid solution was mixed with 0.5 mL of uricase solution. The mixtures were incubated at 37 °C for 20 min. Hydrogen peroxide formation was evaluated by immersing a peroxide test strip into the reaction mixture at time intervals of 0, 5, 10, 15, 20, 25, 30, 35, and 40 min.

### 2.3. Real Sample Preparation and Detection

Human serum samples were collected and stored at −20 °C until analysis. The serum samples were thawed at room temperature and diluted 5-fold with phosphate buffer solution (PBS, pH 7.4) to reduce the possible matrix interference by complex biological components. Spiked recovery assays were carried out by spiking known concentrations of UA into the pre-diluted serum matrix, followed by the enzymatic-plasmonic colorimetric detection cascade as described above in Section 2.2.7.

### 2.4. Green Synthesis of AgNPs

The AgNPs were synthesized by the green route under the optimum conditions as described earlier [49]. In brief, water hyacinth leaf extract (1:1, *v*/*v*) was mixed with AgNO_3_ solution (10 mM), the pH was adjusted to 8.5, and starch was added as a stabilizing agent. The mixture was stirred suitably at 390 rpm. The sample was irradiated with 10 W UV-A (365 nm) light at room temperature for one hour.

According to our earlier mechanistic research, the water hyacinth extract may include active phytoconstituents that contribute to effective synthesis, such as flavonoids, tannins, and polyphenols. These biomolecules donate electrons from the hydroxyl (-OH) groups and help in the reduction of silver ions (Ag^+^) to metallic silver (Ag^0^). Phenolic compounds adsorb on the surface of the newly formed nanoparticles and also act as a capping matrix that stabilizes the AgNPs and prevents their aggregation [49].

### 2.5. Apparatus and Instrumentation

The localized surface plasmon resonance (LSPR) absorption spectra and optical properties of the synthesized AgNPs during the uric acid detection assays were monitored using a UV–Vis spectrophotometer (AvaSpec-EDU, Avantes, Apeldoorn, The Netherlands). All spectral acquisitions were recorded within a scanning wavelength window of 300 to 800 nm using a standard 10 mm path-length quartz cuvette at room temperature. The particle size and shape of the AgNPs were characterized by a JEM-1400 transmission electron microscope (TEM; JEOL Ltd., Tokyo, Japan). The crystalline structure and phase purity of the green-synthesized AgNPs were comprehensively characterized by X-ray diffraction (XRD) on a Bruker D8 Advance diffractometer (Bruker Corp., Billerica, MA, USA). The mechanism of synthesis and specific biomolecular functional groups responsible for the reduction and capping processes were systematically studied by Fourier transform infrared spectroscopy (FTIR) using a PerkinElmer Spectrum 400 FTIR spectrometer (PerkinElmer, Inc., Waltham, MA, USA).

### 2.6. Statistical Analysis

The UV–Vis spectral characterizations, the optimization trials, and the colorimetric biosensing assays were performed in triplicate (N = 3) independently to ensure the reproducibility and analytical fidelity of the experimental measurements. The quantitative data are expressed as the mean value ± standard deviation (SD). Statistical significance and error propagation were evaluated using Microsoft Excel Version 2021 (Microsoft Corp., Redmond, WA, USA) and OriginPro 2024 (OriginLab Corporation, Northampton, MA, USA).

## 3. Results and Discussion

### 3.1. Characterization of AgNPs

**Figure 1:**
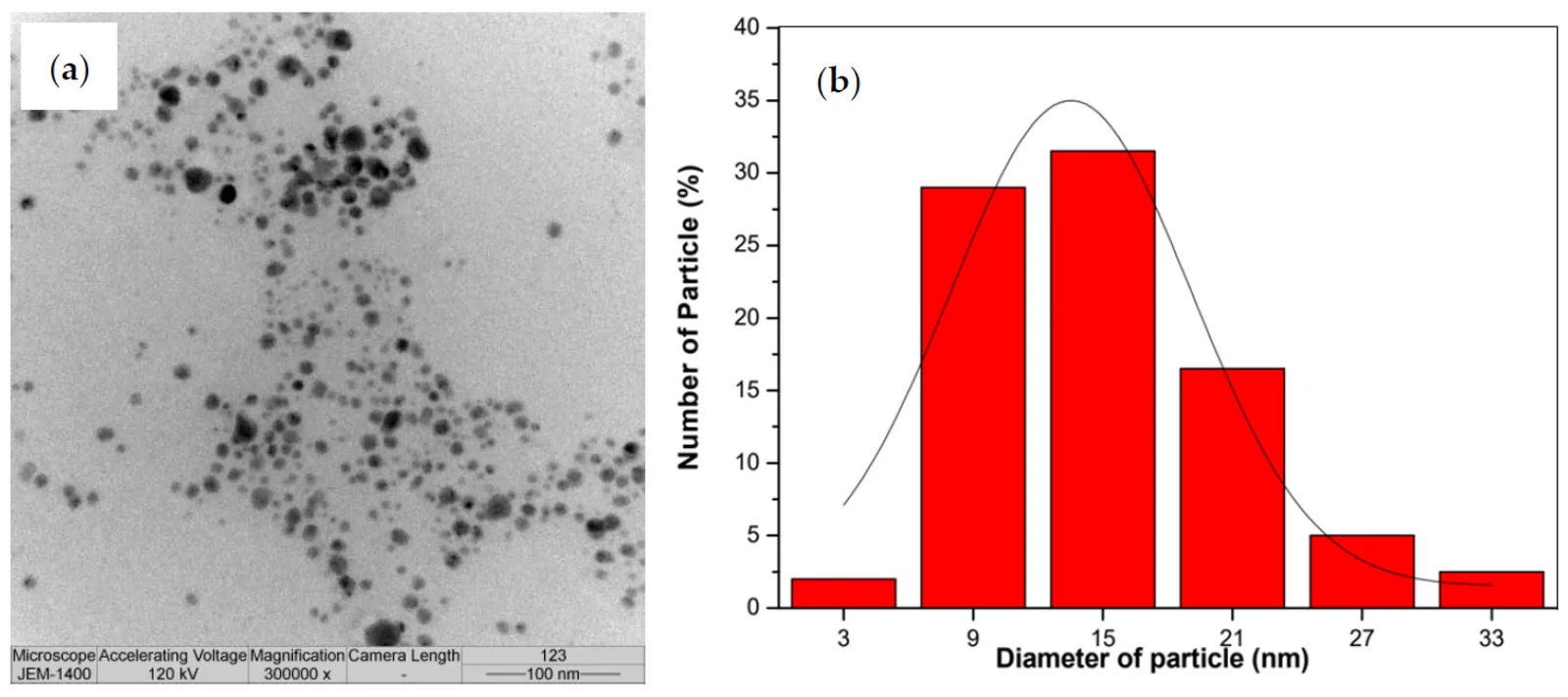
Physical characterization of the AgNPs: (**a**) TEM micrograph; (**b**) corresponding particle size distribution histogram before uric acid detection.

To intuitively evaluate the morphology, dispersion state and precise core size distribution of the green-synthesized AgNPs, a transmission electron microscopy (TEM) analysis was performed, and the results are illustrated in Figure 1. The TEM micrographs (Figure 1a) clearly demonstrate that the synthesized AgNPs exhibit a predominately spherical geometry with a highly uniform and well-defined boundary. Importantly, the nanoparticles show excellent colloidal dispersion with almost no significant agglomeration or clustering noticed in the imaged areas. Such a highly dispersed state serves as direct physical evidence for the successful capping of nanoparticles by the natural biomolecules (like flavonoids, polyphenols, and soluble proteins) available in the leaf extract of *Eichhornia crassipes* (water hyacinth). The development of a protective organic shell around the metallic silver core (Ag^0^) from these biogenic macromolecules provides strong steric and electrosteric repulsion, which effectively prevents spontaneous aggregation.

The corresponding particle size distribution histogram (Figure 1b), measured from the diameters of >100 randomly selected nanoparticles, shows a narrow size distribution from 12 to 17 nm, with the average core diameter calculated to be approximately 15.4 nm. This physical core size obtained from TEM is in excellent agreement with the average crystallite size calculated from the XRD pattern using the Debye–Scherrer equation (~16.8 nm). The slight discrepancy between the crystallite size from XRD and physical size from TEM is to be expected as XRD represents the average size of single crystalline domains and TEM is the actual physical size of individual particles.

Furthermore, the relatively small and uniform size of the AgNPs (below 20 nm) is highly advantageous for colorimetric sensing applications. According to Mie scattering theory [52], nanoparticles in this size range exhibit an exceptionally high surface-to-volume ratio and a strong, sharp localized surface plasmon resonance (LSPR) absorption band centered at approximately 415–430 nm. This high surface reactivity makes the nanoparticles exceptionally sensitive to surface-confined chemical events. Consequently, when exposed to the enzymatic-mediated hydrogen peroxide (H_2_O_2_) generated from the oxidation of uric acid, these small-sized AgNPs undergo highly efficient oxidative etching, leading to rapid particle dissolution and a distinct, easily detectable visual bleaching response.

**Figure 2:**
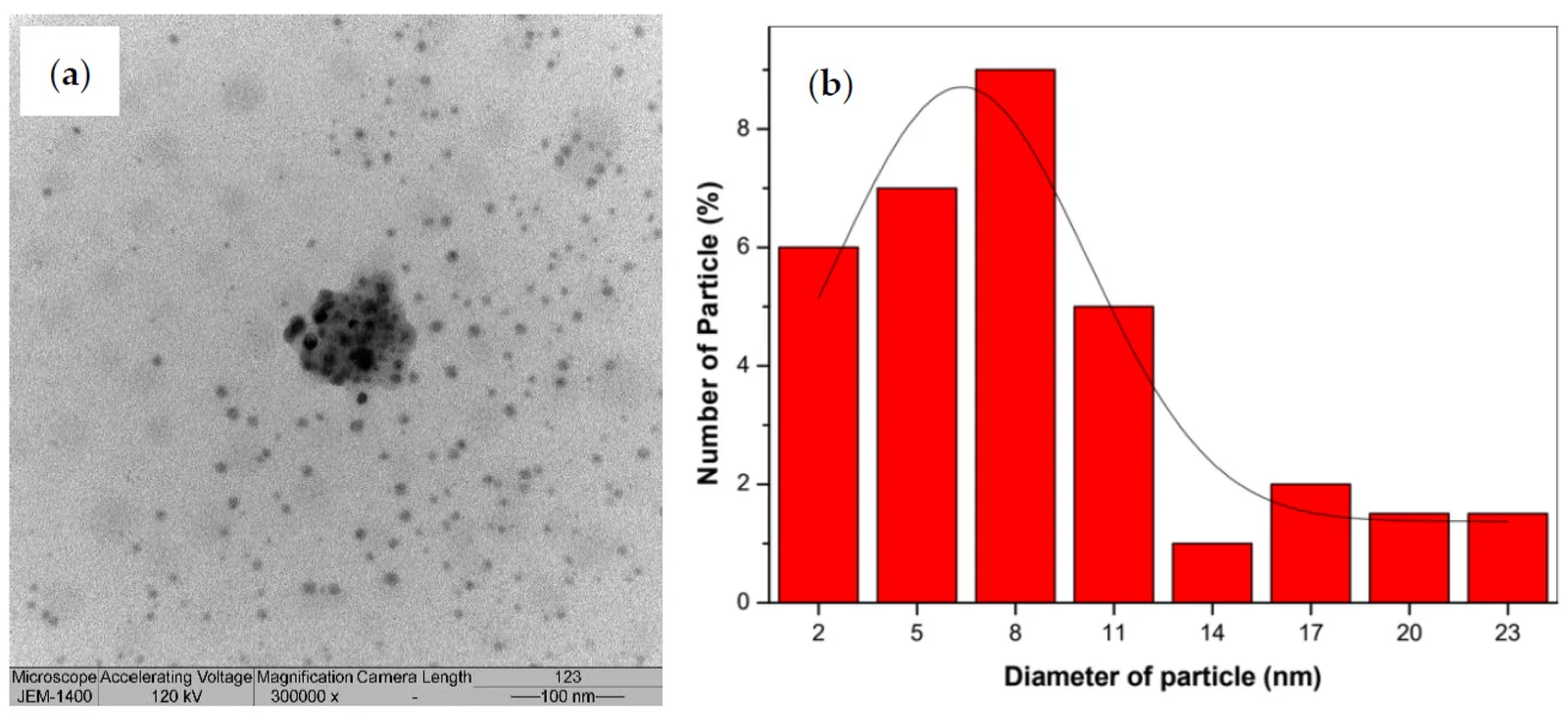
Physical characterization of the AgNPs: (**a**) TEM micrograph; (**b**) corresponding particle size distribution histogram after uric acid detection.

After the colorimetric detection of uric acid, TEM analysis was performed to obtain direct physical evidence of the sensing mechanism and to confirm the structural evolution of the biogenic silver nanoparticles during the assay (Figure 2).

The TEM micrograph after uric acid exposure (Figure 2a) is starkly different from the pristine, highly uniform spherical nanoparticles prior to the reaction. After the sensing process, the AgNPs show severe surface erosion, highly irregular and fragmented boundaries, and a significant degree of particle dissolution. The well-defined structural boundaries of the metallic core (Ag^0^) are visibly degraded with a noticeable population of smaller, sub-nanometer silver clusters and dissolved ionic species (Ag^+^) dispersed throughout the matrix. The extensive physical destruction of the core morphology gives unambiguous, direct evidence of the oxidative etching process. It confirms that the H_2_O_2_, which is stoichiometrically produced from the upstream uricase-catalyzed oxidation of uric acid, successfully penetrates the protective biogenic capping layer, which in turn drives the chemical dissolution of the plasmonic silver cores.

This morphological degradation is quantified further via the corresponding particle size distribution histogram (Figure 2b). Upon measurement of the remaining nanoparticles post-detection, the average physical core diameter exhibits a dramatic reduction, shifting down to approximately 8.2 nm, accompanied by a broader and more irregular distribution profile compared to the pristine state. This sharp decline in core size directly explains the optical macroscopic changes observed in the sensing system. According to Mie scattering theory, the LSPR properties of AgNPs are strictly dependent on particle size, geometry, and structural integrity. The oxidative dissolution and physical shrinkage of the silver cores lead to a catastrophic collapse of the collective electron oscillations, which manifests spectroscopically as a severe dampening of the LSPR absorption peak (approx 420 nm) and macroscopically as the distinct, concentration-dependent visual bleaching (color fading) of the reaction mixture.

**Figure 3:**
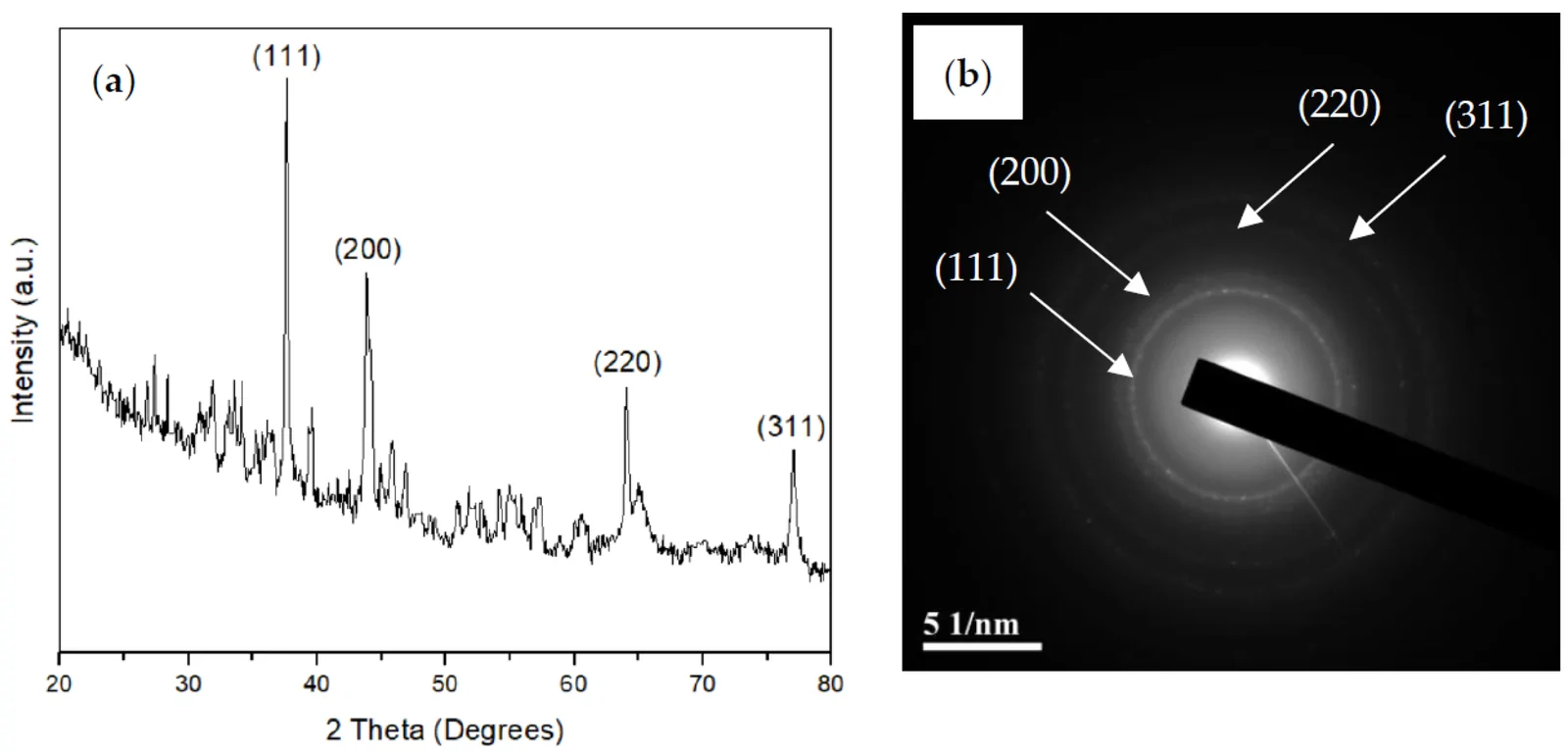
Structural characterization of AgNPs: (**a**) XRD diffraction pattern confirming crystalline structure; (**b**) SAED pattern indicating polycrystalline nature of AgNPs.

The crystalline structure of the synthesized AgNPs was investigated using X-ray diffraction (XRD), and the obtained diffraction pattern is presented in Figure 3. The XRD spectrum exhibited distinct diffraction peaks at 2θ values of approximately 38.1°, 44.3°, 64.5°, and 77.4°, which can be indexed to the (111), (200), (220), and (311) crystallographic planes, respectively. These characteristic reflections correspond to the face-centered cubic (fcc) structure of metallic silver, in agreement with the standard data from the Joint Committee on Powder Diffraction Standards (JCPDS card no. 04-0783).

The presence of these well-defined peaks confirms the successful formation of crystalline AgNPs through the green synthesis process using water hyacinth leaf extract. Among these, the (111) plane exhibited the highest intensity, indicating that the synthesized nanoparticles preferentially grow along this crystallographic orientation, which is commonly observed for silver nanostructures due to its lowest surface energy.

No significant impurity peaks were observed in the XRD pattern, suggesting a high purity of the synthesized AgNPs. However, the presence of a weak and broad background signal may be attributed to the amorphous organic compounds originating from the plant extract, which act as reducing and stabilizing agents during nanoparticle formation.

The average crystallite size of the AgNPs was estimated using the Debye–Scherrer equation:

(1) $$D=\frac{K\lambda }{\beta \cos\theta }$$

where *D* is the crystallite size, *K* is the shape factor (0.9), *λ* is the X-ray wavelength (1.54 Å), *β* is the full width at half maximum (FWHM) of the diffraction peak (0.5°), and *θ* is the Bragg angle. Based on the (111) diffraction peak, the average crystallite size was calculated to be approximately 16.8 nm, indicating the formation of nanoscale crystalline domains.

In our previous report [49], the surface chemistry and functional groups responsible for the reduction and stabilization of the AgNPs using *Eichhornia crassipes* extract were systematically verified by FTIR spectroscopy. Briefly, the prior screening showed prominent vibrational bands associated with polyphenolic O–H stretching, amino acid C=O stretching and conjugated amide linkages, confirming that natural flavonoids and soluble proteins form a dense biogenic capping shell around the Ag cores. In the context of the current sensing application, this pre-established biogenic interface serves a crucial structural property role: it provides robust electrosteric stabilization that effectively shields the AgNPs from non-specific matrix interferences and spontaneous chemical aggregation when exposed to complex biological matrices like human serum, thereby preserving the sensor’s analytical specificity for uric acid detection.

**Figure 4:**
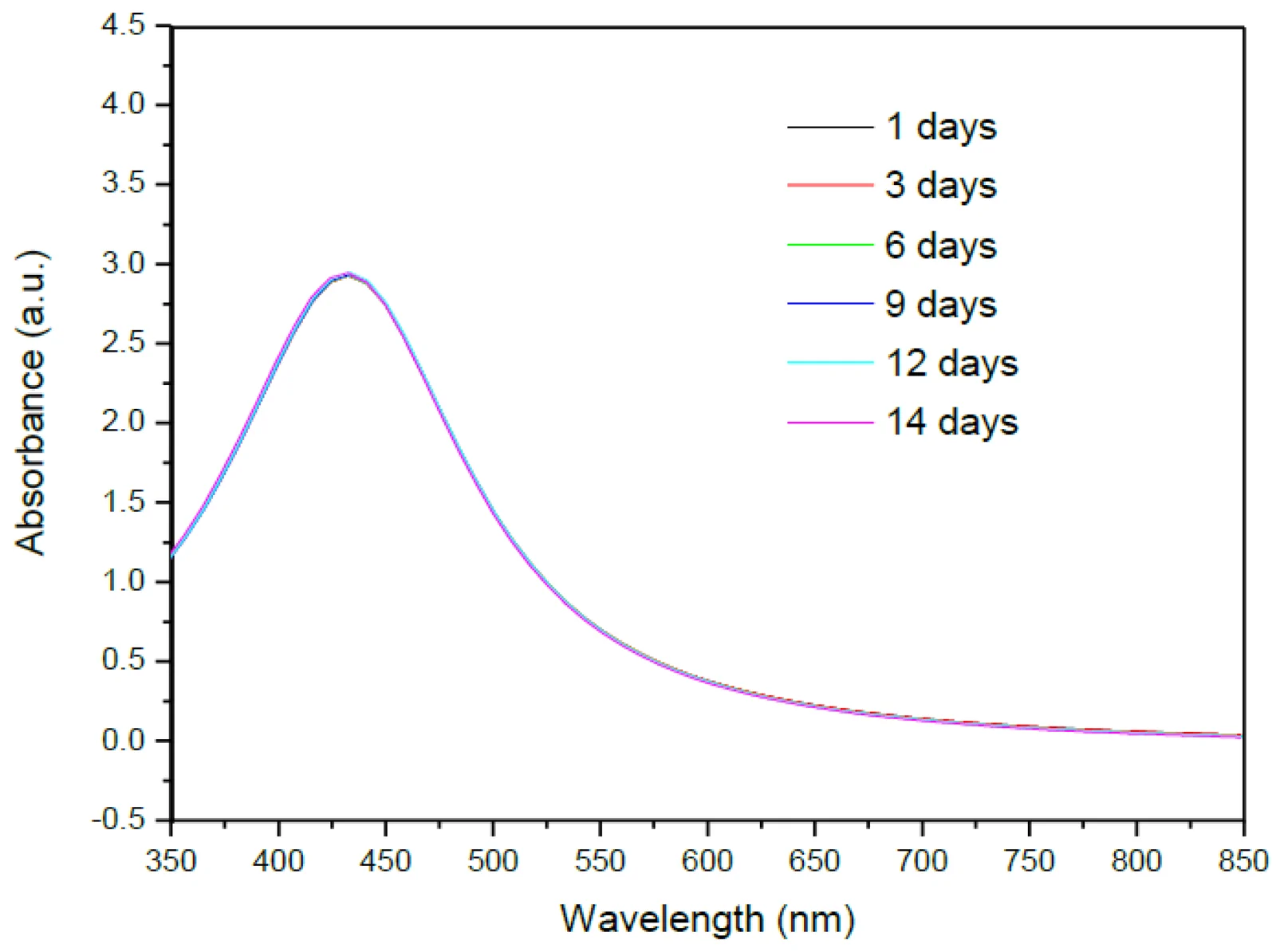
UV–Vis absorbance spectra of the green-synthesized AgNPs over 14 days.

The colloidal robustness and shelf-life of the green-synthesized AgNPs were systematically evaluated during a storage period of 14 days, an important parameter to ensure the reproducibility and accuracy of any downstream colorimetric sensing application. The UV–Vis absorption profiles of the AgNPs were recorded at regular intervals during storage at ambient conditions, as shown in Figure 4.

The colloidal system showed excellent structural and optical stability over the entire 14-day evaluation period. The characteristic LSPR absorption band was highly symmetric and tightly centered at ~420 nm. The preservation of the sharp and well-defined LSPR peak profile over 14 days confirms the fact that the individual AgNP cores remained physically isolated and highly monodisperse in the aqueous medium.

### 3.2. Colorimetric Detection Mechanism of AgNPs

**Figure 5:**
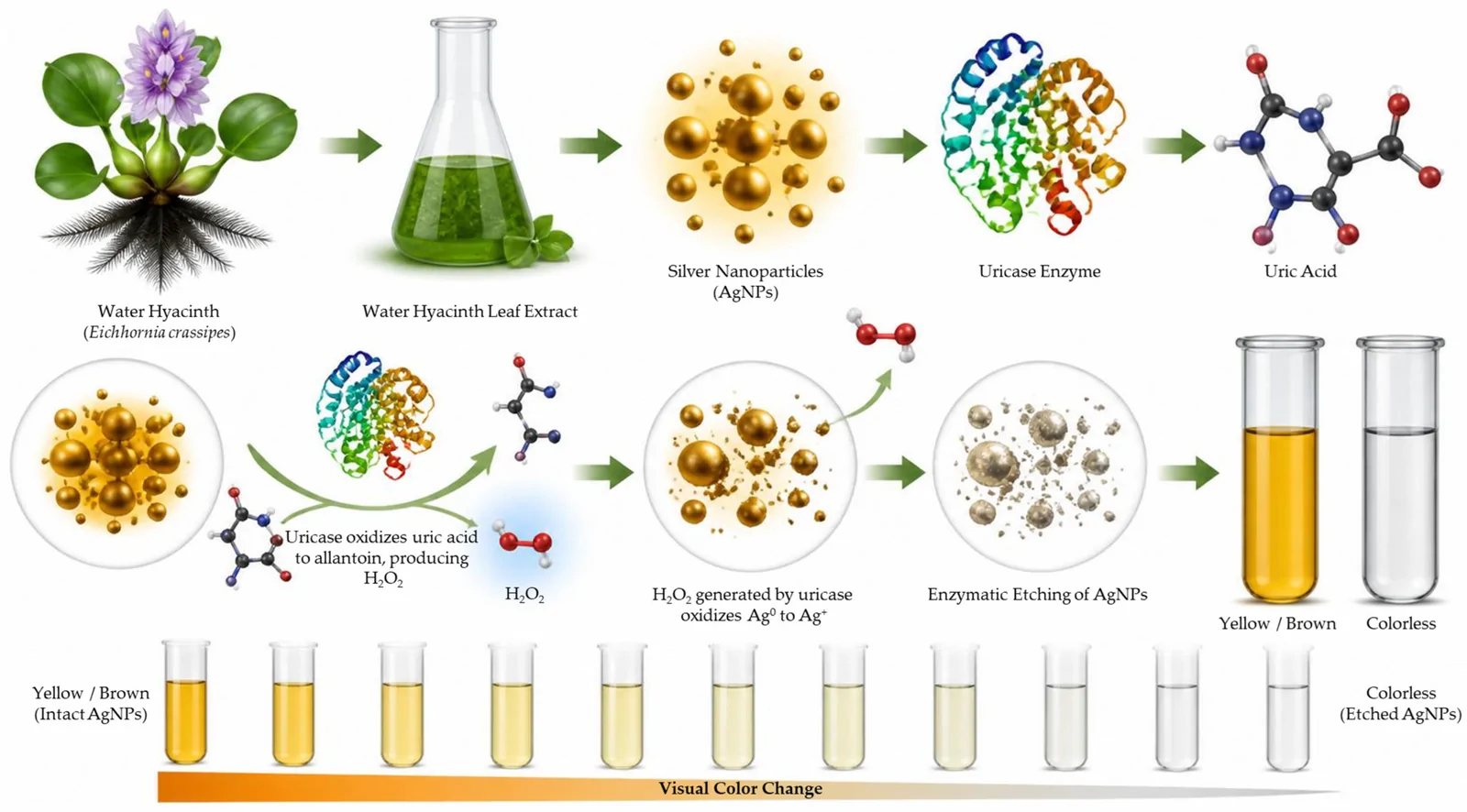
Schematic illustration of the colorimetric sensor for uric acid detection based on the uricase-mediated etching of AgNPs synthesized using water hyacinth leaf extract.

The optical transduction of the developed colorimetric biosensor is based on the cascade process of enzymatic catalysis and subsequent chemical etching (Figure 5). To rule out the possible interferences from endogenous active species in biological matrices (including ascorbic acid, dopamine, and glutathione), the sensor is equipped with a “Dual-Gate” protection design to ensure excellent selectivity for uric acid (UA).

#### 3.2.1. Enzymatic Selectivity

The colorimetric sensing of UA by the developed platform is controlled by a two-step enzymatic cascade reaction. The mechanism combines the highly specific catalytic activity of uricase with the subsequent oxidative dissolution of AgNPs [53].

In the first stage of the cascade, uricase selectively catalyzes the oxidation of UA in the presence of dissolved oxygen and water to generate allantoin, carbon dioxide, and hydrogen peroxide (H_2_O_2_) as a key stoichiometric by-product:

(2) $$Uricacid+O_{2}+2H_{2}O⟶Allantoin+CO_{2}+H_{2}O_{2}$$

In the second stage, the enzymatically generated H_2_O_2_ works as a powerful chemical etchant. It penetrates through the organic capping layer and oxidizes the zero-valent metallic silver core (Ag^0^) of the AgNPs into soluble silver ions (Ag^+^) according to the following redox equation:

(3) $$2Ag^{0}+H_{2}O_{2}+2H^{+}⟶2Ag^{+}+2H_{2}O$$

#### 3.2.2. Biogenic Capping Shield

Even if trace quantities of strong reducing agents attempt to directly react with the colloidal nanoparticles, the biogenic capping layer of the AgNPs offers a strong secondary physical and chemical barrier.

Steric hindrance: Our AgNPs are prepared with the leaf extract of *Eichhornia crassipes* (water hyacinth) as opposed to bare or chemically synthesized nanoparticles with highly exposed metallic surfaces. The dense electrosterically stabilizing shell consists of natural polyphenols, flavonoids, and proteins (see FTIR in Section 3.1) that inhibit larger or highly charged interfering molecules from directly reaching the silver core.

Thermodynamic resistance to reduction: Species such as ascorbic acid, dopamine, and glutathione are thermodynamically strong reducing agents. Their interaction with silver species promotes the reduction of silver ions back to metallic silver (Ag^+^ to Ag^0^) rather than oxidative etching (Ag^0^ to Ag^+^). The sensor signal is based on the destruction of the plasmonic core (etching/bleaching) to generate a signal, meaning that the addition of reducing agents does not help to dampen or blue-shift the LSPR peak. This thermodynamic mismatch means that any non-specific adsorption of reducing agents on the biogenic shell cannot mimic the oxidative etching caused by enzymatic H_2_O_2_.

This synergistic Dual-Gate design (enzymatic selectivity + biogenic capping shield) empowers the sensor to isolate the target signal, which guarantees that the collapse of the LSPR band is a direct and exclusive consequence of uric acid concentration, even when operated in complex matrices.

**Figure 6:**
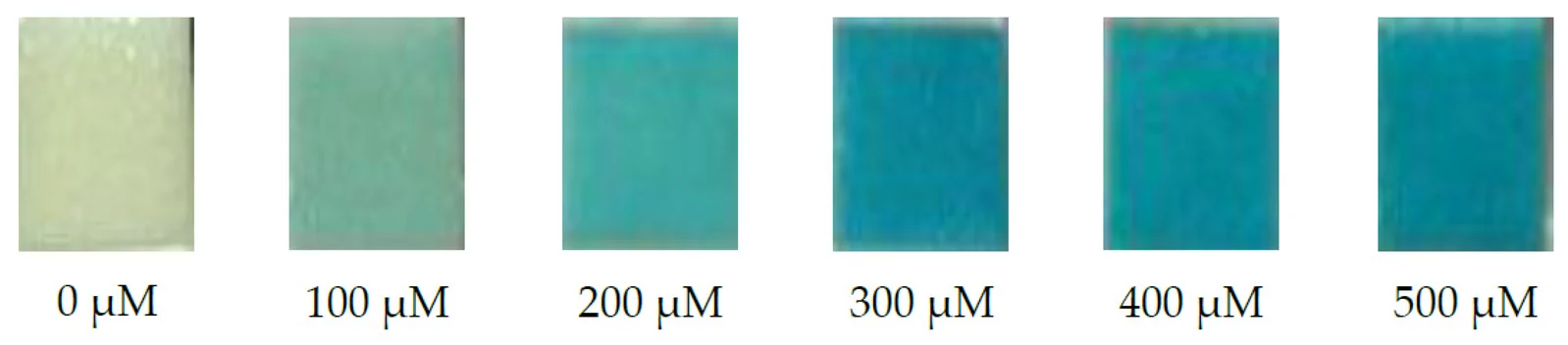
Visual colorimetric assay for H_2_O_2_ quantification derived from the enzymatic reaction between uric acid and uricase at varying initial concentrations (0, 100, 200, 300, 400, and 500 µM).

The experimental determination of the production of H_2_O_2_ in the enzymatic reaction of UA and uricase evidenced a proportional relationship; as the initial concentration of uric acid increases, the yield of generated H_2_O_2_ increases proportionally. Such stoichiometric increase is due to the enzymatic biocatalysis of uric acid by uricase, which is selective to the target analyte into allantoin and H_2_O_2_ as co-products. As a consequence, the linear increase of H_2_O_2_ production provides the quantitative driving force needed for the concentration-dependent oxidative etching of the AgNPs, establishing a robust correlation between UA concentration and the observed colorimetric signal decay (Figure 6).

This relationship is critical for the development of a quantitative sensing platform, as the produced H_2_O_2_ acts as a key intermediate responsible for the oxidation of AgNPs by H_2_O_2_, which will reduce their concentration [54,55,56].

The generated H_2_O_2_ interacts with the AgNPs’ surface, which causes a change in the LSPR of AgNPs that can be seen as a change in the intensity of absorbance. This means that the oxidation of AgNPs due to H_2_O_2_ reduced the concentration of AgNPs and reduced the intensity of LSPR.

**Figure 7:**
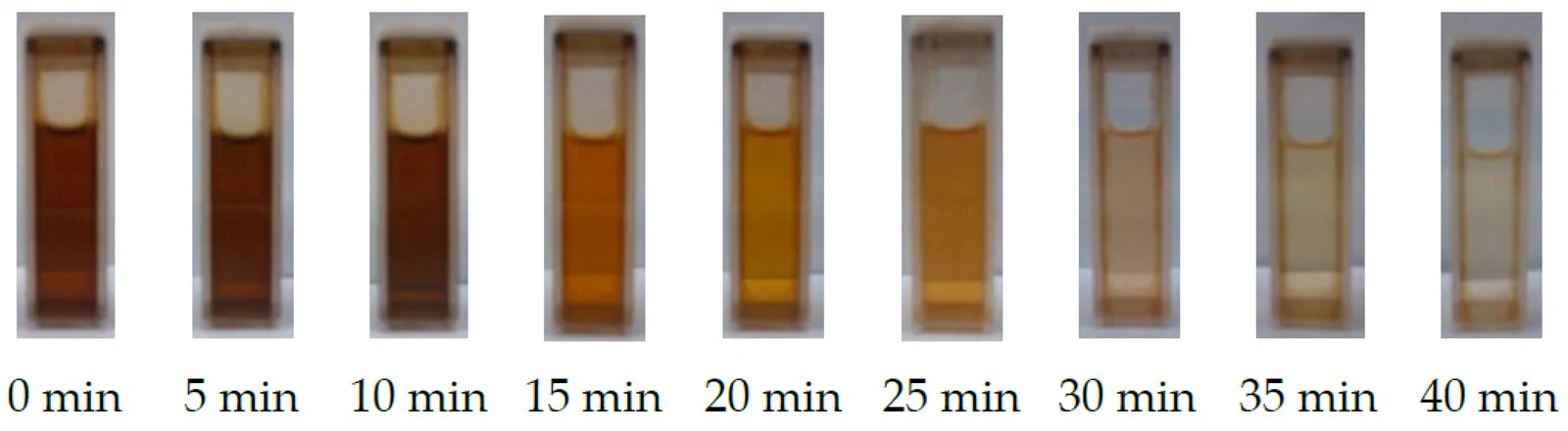
Color change of AgNPs etched by H_2_O_2_ at 100 µM H_2_O_2_ concentration over time.

This oxidative etching process leads to a gradual decrease in the average particle size and the overall concentration of intact nanoparticles in the system. Therefore, the LSPR of the AgNPs collapses, which is macroscopically reflected as a distinct color bleaching effect, changing from a deep brown color to a pale yellow or a completely colorless state, as shown in Figure 7.

Although AgNPs are capped and stabilized by natural biomolecules such as flavonoids, polyphenols, and soluble proteins present in the *Eichhornia crassipes* extract, natural shells provide electrosteric stabilization against spontaneous chemical aggregation. However, the small, uncharged H_2_O_2_ molecules can easily penetrate this biogenic capping shield to interact directly with the metallic silver surface, rendering the sensor highly sensitive and specific toward UA concentrations.

#### 3.2.3. Temporal Evaluation of Oxidative Etching Kinetics

The time-dependent etching kinetics and optimization of the analytical response of the sensing platform were investigated by systematically monitoring the visual colorimetric changes of the AgNPs at different time points (0, 5, 10, 15, 20, 25, 30, 35, and 40 min) in the presence of a representative 100 µM concentration of H_2_O_2_ (Figure 7). At the beginning of the reaction (0 min), the sensing mixture retained its pristine, intense deep brown color, which was associated with a strong LSPR absorption maximum and indicated that the nanoparticles remained fully intact. After the addition of H_2_O_2_ (5 to 15 min), a gradual transition to a lighter copper-brown hue was observed, indicating the initial surface erosion of the metallic core by the diffusing etchant molecules. Between 20 and 30 min, the reaction proceeded rapidly, resulting in a highly visible bleaching of the solution into a faint brownish-yellow color, which corresponds to a substantial contraction of the AgNP core sizes and a parallel decrease in the density of LSPR-active centers. Eventually, the solution faded into an extremely pale yellow, approaching a near-colorless state at 40 min, representing the attainment of a chemical steady state wherein the vast majority of the Ag^0^ had been oxidized into soluble Ag^+^ ions. The time profile indicates that an incubation time of 30–40 min is highly optimal for obtaining a distinct, stable, and reproducible color change, providing excellent visual contrast for reliable naked-eye point-of-care testing (POCT) and semi-quantitative analysis without sophisticated spectroscopic instrumentation.

**Figure 8:**
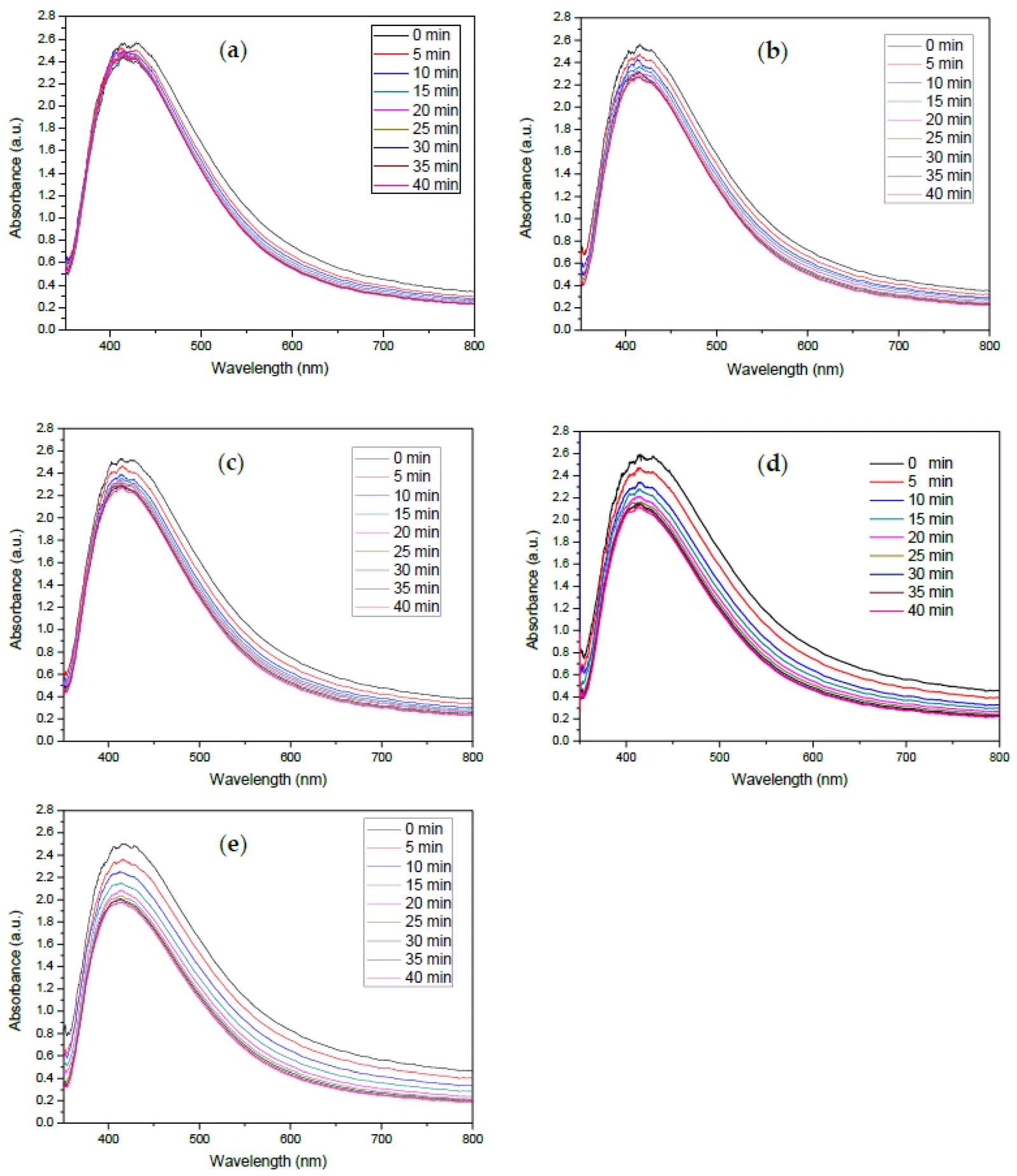
UV–Vis absorbance spectra of AgNPs with uric acid at various H_2_O_2_ concentrations: (**a**) 100 µM; (**b**) 150 µM; (**c**) 200 µM; (**d**) 300 µM; (**e**) 500 µM, recorded at different time intervals.

The temporal UV–Vis absorption profiles in Figure 8 clearly demonstrate the proposed oxidative etching mechanism [53]. Standard nanoparticle aggregation is strictly characterized by the appearance of a new, red-shifted LSPR band (typically at >550 nm) and a subsequent color transition to purple/grey due to interparticle plasmon coupling. In contrast, the spectra in Figure 8 exhibit a progressive, monotonic decrease in the primary LSPR peak intensity (approx 420 nm) with a subtle blue-shift, and absolutely no emergent absorption bands at longer wavelengths. Mie scattering theory predicts that this LSPR damping and blue-shift are direct evidence of systematic nanoparticle size reduction and decrease in particle concentration [52]. This optical behavior confirms that the H_2_O_2_ produced from the uric acid–uricase reaction serves only as a chemical etchant, causing the stoichiometric dissolution of zero-valent Ag^0^ into non-plasmonic Ag^+^ and not promoting physical particle aggregation.

### 3.3. Time-Dependent Optical Response

**Figure 9:**
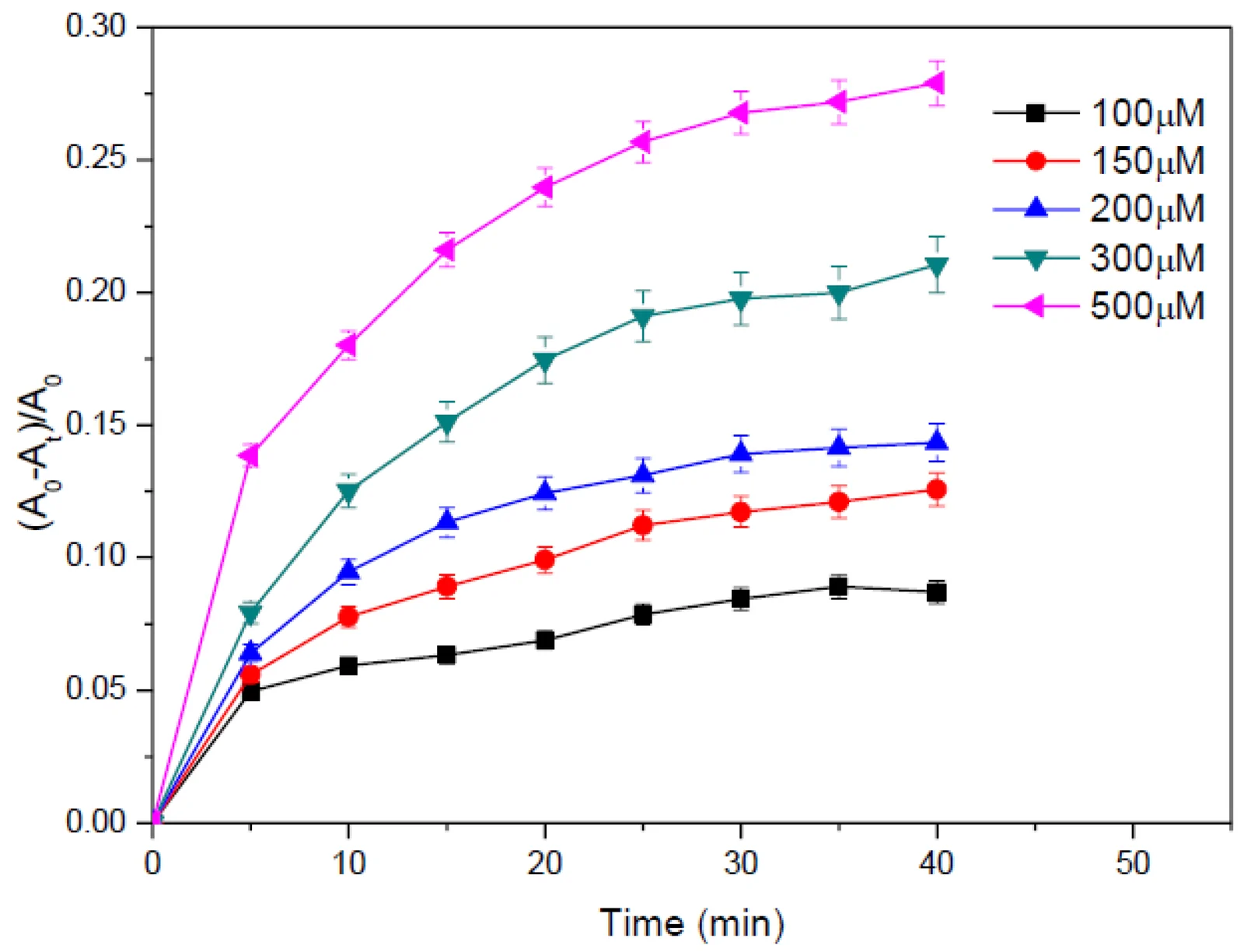
Time-dependent UV–Vis absorbance spectra of AgNPs in the presence of uric acid at varying concentrations: (a) 100 µM; (b) 150 µM; (c) 200 µM; (d) 300 µM; (e) 500 µM.

The time-dependent optical response of the synthesized AgNPs in the presence of uric acid was investigated to evaluate the reaction kinetics and signal stability of the proposed sensing system. The evolution of the UV–Vis absorbance spectra over time at different uric acid concentrations is presented in Figure 9.

Upon the introduction of uric acid and uricase, a gradual change in the absorbance spectra of AgNPs was observed as a function of time. This behavior is attributed to the continuous generation of H_2_O_2_ through the enzymatic oxidation of uric acid, which subsequently induces oxidative modification of the AgNPs surface. As the reaction proceeds, the absorbance intensity at the characteristic plasmonic peak (415–430 nm) progressively decreases, indicating a time-dependent modulation of the LSPR properties.

At higher uric acid concentrations, the rate of absorbance decrease was significantly faster, suggesting enhanced production of H_2_O_2_ and accelerated surface etching of the AgNPs. This concentration-dependent kinetic behavior confirms that the sensor response is governed by the enzymatic reaction rate and subsequent nanoparticle transformation. In contrast, at lower concentrations, the spectral changes occurred more gradually, reflecting slower reaction kinetics.

The time-resolved absorbance profiles further reveal that the system reaches a quasi-equilibrium state after a certain incubation period, beyond which minimal changes in absorbance are observed. This indicates the completion of the enzymatic reaction or stabilization of the nanoparticle system. The determination of this optimal reaction time is crucial for achieving consistent and reproducible analytical measurements.

The reaction reached a steady-state signal within 10 min, which was selected as the optimal detection time for subsequent analysis.

### 3.4. Performance Analysis

**Figure 10:**
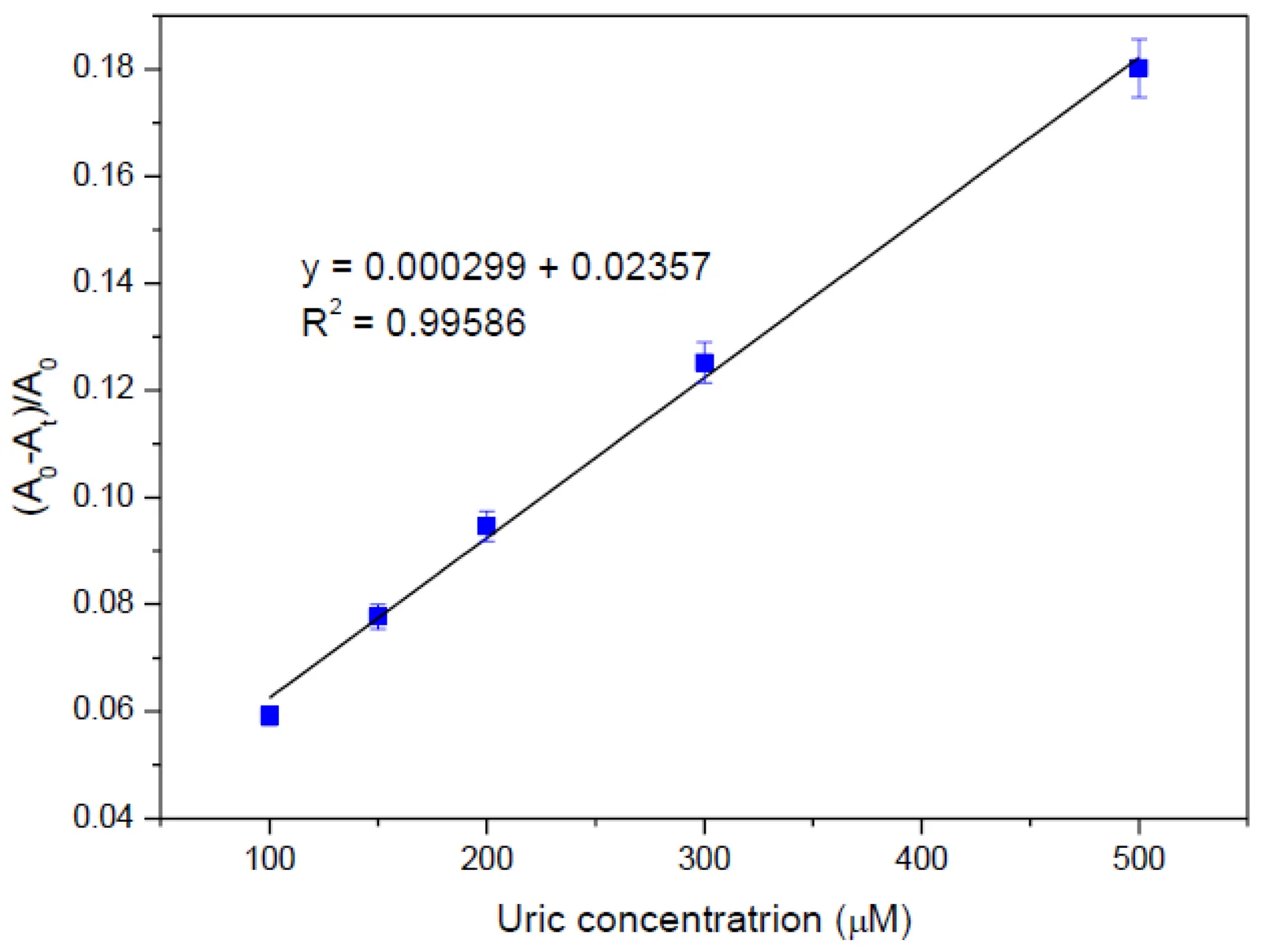
Calibration curve for uric acid detection based on the relative change in absorbance (ΔA) of AgNPs as a function of uric acid concentration.

The analytical performance of the developed AgNPs-based colorimetric sensor for UA detection was systematically evaluated in terms of sensitivity, linearity, LOD, and reproducibility. The calibration curve was constructed by plotting the relative change in absorbance (ΔA) of the AgNPs as a function of uric acid concentration, as shown in Figure 10.

A good linear relationship between the absorbance response and uric acid concentration was obtained over the range of 100–500 µM, with a correlation coefficient (R^2^ = 0.99), indicating excellent linearity within the clinically relevant concentration range. The slope of the calibration curve reflects the sensitivity of the sensing platform, demonstrating the capability of the AgNPs to effectively transduce chemical signals into measurable optical responses.

The LOD [51], calculated based on the 3σ/slope criterion, was determined to be 25.89 µM, suggesting that the proposed sensor is sufficiently sensitive for practical uric acid detection in biological samples. This detection limit is comparable to previously reported colorimetric sensors based on plasmonic nanomaterials.

In addition to sensitivity, the repeatability of the sensor was assessed by performing multiple measurements under identical conditions. The RSD was found to be less than 5%, confirming good precision and reproducibility of the system.

The limit of quantification (LOQ) [51], defined as 10σ/slope, was calculated to be approximately 86.29 µM.

Table 1 compares the analytical performance of the proposed AgNPs-based colorimetric sensor with previously reported methods for UA detection. Although some reported techniques exhibit lower limits of detection, many of those systems rely on complex fabrication procedures, expensive instrumentation, or chemically synthesized nanomaterials. In contrast, the present work employs a simple and environmentally friendly green synthesis approach using water hyacinth leaf extract, enabling sustainable production of AgNPs without the use of hazardous reducing agents.

The developed sensing platform demonstrated satisfactory analytical performance, including a linear detection range of 100–500 µM, a LOD of 25.89 µM, and good reproducibility with an RSD below 5%. Importantly, the working range covers clinically relevant uric acid concentrations in human serum, while recovery values between 96.3% and 103.4% confirm the reliability of the method in real biological samples.

These results indicate that the proposed sensor achieves a favorable balance between analytical performance, operational simplicity, and environmental sustainability. Therefore, the developed platform represents a promising alternative to conventional uric acid sensing methods, particularly for low-cost and practical biosensing applications.

The proposed method also avoids complicated electrode modification and sophisticated instrumentation required in electrochemical or fluorescence-based systems.

A comprehensive evaluation against previously reported AgNP-based colorimetric sensors is summarized in Table 1. Admittedly, the proposed sensing system possesses a higher LOD (25.89 µM) relative to several chemical-reduction strategies or core-shell nano-assemblies [53,57,58,59,60]. Nevertheless, this detection threshold remains significantly lower than the standard human physiological and clinical cutoff levels for uric acid in serum (which typically exceed 150 µM for hypouricemia and range up to 420 µM for normal limits). Consequently, the current sensitivity is fully capable of distinguishing healthy states from clinical hyperuricemia. Crucially, while most existing systems heavily rely on hazardous synthetic capping agents, multi-step chemical functionalization, or high-energy hydrothermal synthesis [61,62,63,64] that often suffer from severe bio-fouling or chemical interferences, our platform introduces a waste-to-wealth sustainable paradigm. By capitalizing on the natural biogenic capping shield from *Eichhornia crassipes* via a rapid, UV-assisted green pathway, this system bypasses toxic precursors entirely. It delivers an unmatched cost-effective, easily operating, and portable (naked-eye resolvable) point-of-care testing (POCT) device uniquely optimized for highly accurate quantification in complex human serum matrices.

From a translational and clinical biotechnology perspective, the developed *Eichhornia crassipes*-mediated AgNP colorimetric sensor has many serious practical advantages over standard laboratory-based diagnostic modalities for uric acid determination such as high-performance liquid chromatography (HPLC) and automated enzymatic colorimetric analyzers.

**Figure 11:**
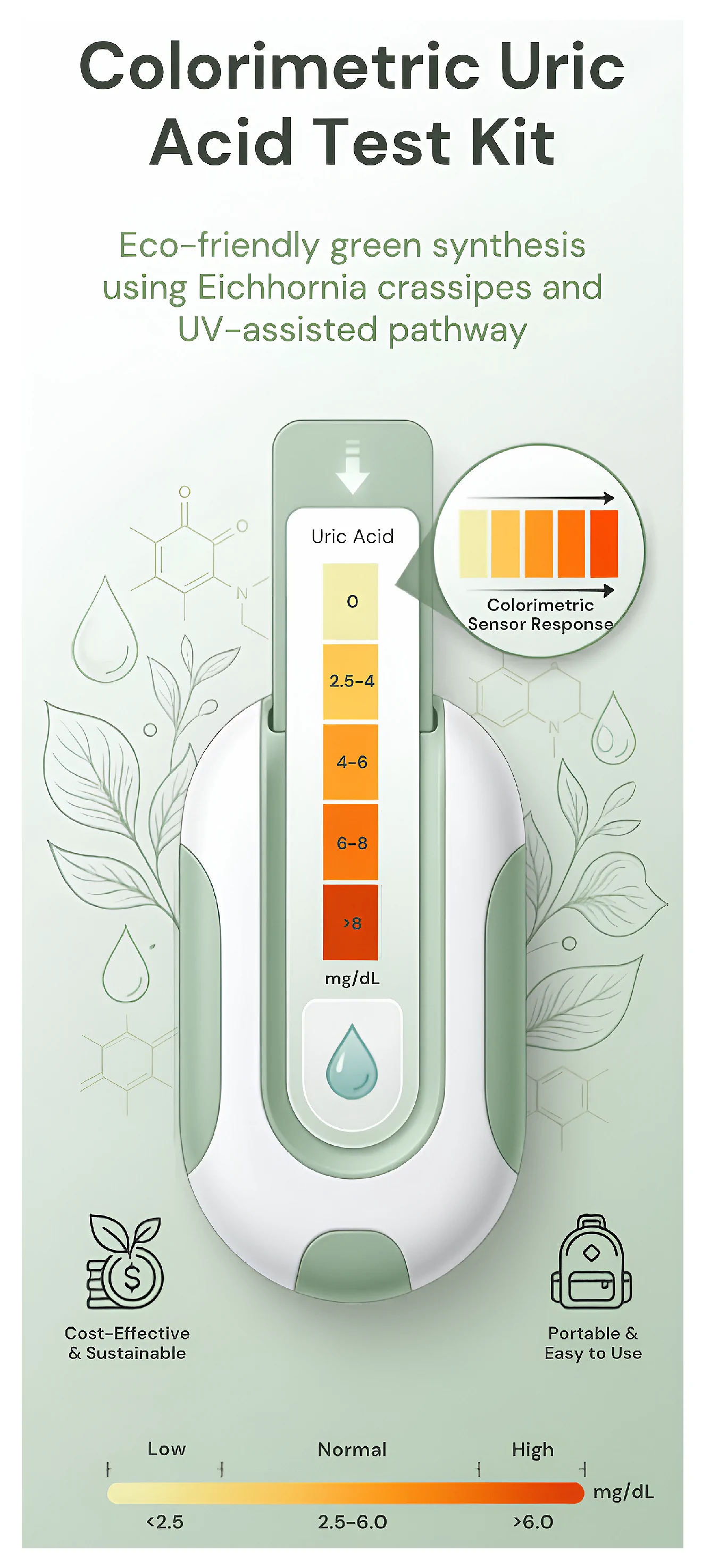
Colorimetric sensor test kit for uric acid detection.

First and foremost is its extraordinary operational simplicity and instrument-free readability. The conventional clinical assays usually need sophisticated instrumentation, trained technicians and tedious sample pretreatment procedures, in stark contrast to our proposed sensing platform relying on a highly intuitive chemical-to-optical transduction mechanism. The concentration-dependent oxidative etching of the plasmonic silver cores provides a distinct visual bleaching profile that can be readily quantified with a simple UV–Vis spectrophotometer or evaluated semi-quantitatively with the naked eye using a standardized color reference chart. This makes the sensor an exceptional candidate for integration into decentralized POCT devices and self-diagnostic test kits. The developed colorimetric sensor test kit for uric acid detection is illustrated in Figure 11.

Secondly, the platform is characterized by excellent cost-effectiveness and ecological sustainability, which are derived from the principles of green chemistry. The dependence on expensive and hazardous chemical reductants (e.g., sodium borohydride) is totally abrogated by using an invasive and problematic aquatic weed (*Eichhornia crassipes*) as the main reducing and stabilizing agent. This results in a drastic reduction in the cost of material synthesis and mitigation of hazardous waste generation. In addition, the sturdy biogenic capping layer, which is intrinsically deposited on the AgNP surface, imparts superb long-term colloidal stability, thus extending the shelf-life of the test kit up to 14 days under ambient conditions without the need for rigorous refrigeration.

The sensor’s biological “Dual-Gate” selectivity architecture guarantees excellent diagnostic reliability. While it is a simple mixing assay, the strict lock-and-key specificity of uricase, together with the electrosteric barrier provided by the plant-derived organic shell, effectively shields the metallic core from potential false-positive interferences by strong reducing agents (ascorbic acid, glutathione, dopamine) which are routinely found in human serum matrices. This green-engineered colorimetric platform thus enables rapid, low-cost, and reliable screening of uric acid, which holds great promise for early clinical monitoring of hyperuricemia and gout in resource-poor or remote areas.

### 3.5. Real Sample Analysis

The practical usability of the developed AgNPs-based colorimetric sensor was further evaluated by real sample analysis with spiked human serum samples. To mitigate matrix effects (i.e., the non-specific chemical or physical interference of background endogenous components such as proteins, lipids, and unrelated metabolites on the sensor transducer), the blood samples were properly diluted with a buffer solution prior to analysis. In particular, sample dilution is required to reduce the light scattering effects and turbidity resulting from the inherent opaqueness and macromolecular composition of biological sera, which could interfere with the accurate monitoring of the LSPR spectra of the AgNPs. Moreover, dilution preserves the co-existing reducing agents (such as ascorbic acid, glucose, or bilirubin) and renal biomarkers (such as urea and creatinine), which are typically elevated in pathological states, well below the threshold to scavenge the reaction intermediate (H_2_O_2_) or modify the surface of the nanoparticle.

Although the human serum samples were diluted by 5 times to minimize possible matrix interferences, the decrease in analyte concentration did not affect the detection capability of the biosensor. The baseline endogenous UA concentration in the human serum sample was determined to be 216.9 ± 1.2 µM, far within the normal human physiological reference range, and the 5-fold dilution leads to a working concentration of about 43.38 µM in the reaction mixture, which is far above the sensor limit of detection (LOD = 25.89 µM) and within the optimized linear dynamic range, confirming the operational robustness and practical feasibility of the platform for real blood analysis.

The final recovery values were obtained by measuring the baseline uric acid concentration of the unspiked, diluted human serum sample using our proposed colorimetric sensor platform. Then, this endogenous value was subtracted from the total concentration obtained from the spike to calculate the actual “Found” concentration of the spiked analyte. The % Recovery was then calculated by using the standard addition formula:

(4) $$\%Recovery=\frac{C_{Total}-C_{Endogenous}}{C_{Spiked}}\times 100$$

C_Total_ is the measured concentration after spiking, C_Endogenous_ is the baseline concentration that was originally present in the serum and C_Spiked_ is the known added concentration.

To further confirm the analytical reliability and clinical applicability of the developed biosensor, recovery studies in human serum samples were performed by the standard addition method. The concentration of endogenous UA in the unspiked serum matrix was determined as 216.9 ± 1.2 µM (Table 2). This well-defined concentration is in excellent agreement with the physiological reference range of normal humans, and the low standard deviation (SD = 1.2 µM) further reflects the excellent stability and baseline reproducibility of our detection platform. The spiked samples with different target concentrations (100 to 500 µM) showed excellent recovery values (96.3% to 103.4%) along with low relative standard deviations (% RSD ≤ 3.2%). These statistically significant data provide strong evidence that the developed biosensor is immune to complex matrix interferences, delivering high accuracy and precision for real-life blood analysis. The combination of such a protective dilution concept with the high enzymatic selectivity of uricase endows the sensor with excellent analytical fidelity as reflected by the satisfactory recoveries (96.3–103.4%) in Table 2.

The successful detection of uric acid in serum samples highlights the robustness of the proposed system and its potential for practical use in clinical diagnostics. The integration of green-synthesized AgNPs with enzymatic specificity enables a simple, cost-effective, and environmentally friendly approach for uric acid determination.

## 4. Conclusions

In this work, the green synthesis approach was successfully applied to develop a colorimetric sensing platform based on AgNPs for UA detection using water hyacinth leaf extract as a sustainable reducing and stabilizing agent. The synthesized AgNPs exhibited a well-defined crystalline structure and stable optical properties for their effective use as plasmonic signal transducers in an uricase-based enzymatic system. The mechanism of H_2_O_2_-mediated oxidative etching responsible for the analytical signal transduction was unambiguously confirmed by direct morphological evolution and particle dissolution via transmission electron microscopy (TEM).

Importantly, the platform displayed excellent selectivity and matrix tolerance in complex biological environments, due to the synergistic “Dual-Gate” protection system with strict upstream uricase specificity and strong downstream biogenic capping shield. This organic interface efficiently prevented non-specific direct attacks of strong endogenous reducing agents (e.g., ascorbic acid and glutathione), giving rise to excellent repeatability (RSD < 5%) and long-term colloidal stability over 14 days without the assistance of artificial stabilizers.

These results suggest that the proposed method is well suited for real-sample analysis and has great potential for practical use in decentralized clinical diagnostics and point-of-care screening. This work highlights the successful integration of waste biomass valorization with plasmonic nanomaterial-based biosensing, representing a simple, cost-effective, and environmentally friendly alternative to conventional sensing approaches. Thus, the developed platform not only contributes to sustainable nanotechnology, but also provides a promising matrix-tolerant strategy for the development of next-generation colorimetric biosensors.

## Figures and Tables

**Table 1:** Comparison of the performance of the proposed green-synthesized AgNPs-based sensor with previously reported uric acid detection methods.

Materials	Linear Range	LOD	Synthesis Method	Cost & Portability	Real Sample Matrix	Ref.
Silver Nanoprisms	1–40 µM	0.7 µM	Chemical reduction	Standard cost/ Lab-bound	Human urine	[53]
Silver Oxide Doped AC	0.001–0.36 µM	0.207 nM	Chemical reduction	High cost/Lab-bound	Human urine	[57]
Silver Nanoparticles by HAuCl_4_	0.1 nM to 0.1 mM	30 pM	Chemical reduction	High cost/Complex	Human urine	[58]
Silver Nanoparticles-Reduced Graphene Oxide	10–130 µM	1 µM	Chemical reduction	High cost/Toxic surfactant	Diluted human urine	[59]
Ag/Au Nanorods	0.1–1 µM	0.065 µM	Chemical reduction	Complex/Multi-step	Human urine & serum	[60]
Biohybrid Nanosystem	5–1000 µM	1.6 µM	In-vivo biosynthetic	High energy/Cultivation	Human serum	[61]
Sawdust-Deposited Silver Nanoparticles	0.04–0.36 µM	0.004 µM	Semi-green/ Biomass	Low cost/Solid substrate	Pure standard only	[62]
Silver Nanoparticles/N-CQDs	2–60 µM	0.53 µM	Hydrothermal + Chemical	High energy/Complex	Human serum	[63]
Cu_2_O@Ag Nanocomposites	0.5–100 µM	0.4 µM	Self-assembly method	High cost/Multi-step	Human serum	[64]
Biogenic AgNPs (Water Hyacinth)	100–500 µM	25.89 µM	UV-assisted Green (Eco-friendly/No toxic chemical)	Extremely Low cost & On-site POCT	Human Serum	This work

**Table 2:** Recovery results for uric acid determination in spiked human serum samples (N = 3).

Sample	Endogenous UA (µM)	Added UA (µM)	Total Found (µM)	Net Found (µM)	Recovery (%)	RSD (%)
Serum 1	216.9 ± 1.2	100	313.2 ± 3.3	96.3	96.3	3.2
Serum 2	216.9 ± 1.2	200	422.3 ± 5.8	205.4	102.7	2.8
Serum 3	216.9 ± 1.2	300	515.6 ± 7.5	298.7	99.6	2.5
Serum 4	216.9 ± 1.2	500	733.9 ± 16.0	517.0	103.4	3.1

## Data Availability

The original contributions presented in this study are included in the article. Further inquiries can be directed to the corresponding author.

## References

[1] Roman Y.M. (2023). The Role of Uric Acid in Human Health: Insights from the *Uricase* Gene. J. Pers. Med..

[2] Cheng J., Su Y., Wu Y., Zhu L., Chen L., Chang S., Huang K., Xu W. (2025). Uric acid metabolism regulation mechanism by probiotics and prebiotics: From a new perspective. Trends Food Sci. Technol..

[3] Ali D.K., Qader H.A., Fakhre N.A. (2025). Simultaneous analysis of creatinine and uric acid in human urine samples using derivative spectrophotometric techniques as a green analytical protocol. Green Anal. Chem..

[4] Kimura Y., Tsukui D., Kono H. (2021). Uric Acid in Inflammation and the Pathogenesis of Atherosclerosis. Int. J. Mol. Sci..

[5] Yu W., Xie D., Yamamoto T., Koyama H., Cheng J. (2023). Mechanistic insights of soluble uric acid-induced insulin resistance: Insulin signaling and beyond. Rev. Endocr. Metab. Disord..

[6] Borghi C., Rosei E.A., Bardin T., Dawson J., Dominiczak A., Kielstein J.T., Manolis A.J., Perez-Ruiz F., Mancia G. (2015). Serum uric acid and the risk of cardiovascular and renal disease. J. Hypertens..

[7] Chen H., Cai S., Luo J., Liu X., Ou L., Zhang Q., Liedberg B., Wang Y. (2024). Colorimetric biosensing assays based on gold nanoparticles functionalized/combined with non-antibody recognition elements. TrAC Trends Anal. Chem..

[8] Timsans J., Palomäki A., Kauppi M. (2024). Gout and Hyperuricemia: A Narrative Review of Their Comorbidities and Clinical Implications. J. Clin. Med..

[9] Chen Y., Yang J., Rao Q., Wang C., Chen X., Zhang Y., Suo H., Song J. (2023). Understanding Hyperuricemia: Pathogenesis, Potential Therapeutic Role of Bioactive Peptides, and Assessing Bioactive Peptide Advantages and Challenges. Foods.

[10] Zhang W. (2025). Hyperuricemia: Current State and Prospects. Explor. Res. Hypothesis Med..

[11] Lanaspa M.A., Andres-Hernando A., Kuwabara M. (2020). Uric acid and hypertension. Hypertens. Res..

[12] Corey-Bloom J., Haque A., Aboufadel S., Snell C., Fischer R.S., Granger S.W., Granger D.A., Thomas E.A. (2020). Uric Acid as a Potential Peripheral Biomarker for Disease Features in Huntington’s Patients. Front. Neurosci..

[13] Ferraro P.M., Curhan G.C. (2017). Serum Uric Acid and Risk of Kidney Stones. Am. J. Kidney Dis..

[14] Ruocco G., Palazzuoli A. (2019). Hyperuricemia in US Population with Heart Failure: Causal or Incidental Bystander?. Cardiorenal Med..

[15] Shahin L., Patel K.M., Heydari M.K., Kesselman M.M. (2021). Hyperuricemia and Cardiovascular Risk. Cureus.

[16] Du L., Zong Y., Li H., Wang Q., Xie L., Yang B., Pang Y., Zhang C., Zhong Z., Gao J. (2024). Hyperuricemia and its related diseases: Mechanisms and advances in therapy. Signal Transduct. Target. Ther..

[17] Singh S.K., Singh R., Singh S.K., Iquebal M.A., Jaiswal S., Singh A. (2022). Prevalence of Hyperuricemia and the Relationship Between Serum Uric Acid and Hypertension in New Onset Diabetic Patients: A Cross-Sectional Indian Study. Diabetes Metab. Syndr. Obes..

[18] Padda J., Khalid K., Padda S., Boddeti N.L., Malhi B.S., Nepal R., Cooper A.C., Jean-Charles G. (2021). Hyperuricemia and Its Association With Ischemic Stroke. Cureus.

[19] Zuo T., Liu X., Jiang L., Mao S., Yin X., Guo L. (2016). Hyperuricemia and coronary heart disease mortality: A meta-analysis of prospective cohort studies. BMC Cardiovasc. Disord..

[20] Padda J., Khalid K., Almanie A.H., Hennawi H.A., Mehta K.A., Fernando R.W., Padda S., Cooper A.C., Jean-Charles G. (2021). Hyperuricemia in Patients With Coronary Artery Disease and Its Association With Disease Severity. Cureus.

[21] Gonçalves D.L.N., Moreira T.R., da Silva L.S. (2022). A systematic review and meta-analysis of the association between uric acid levels and chronic kidney disease. Sci. Rep..

[22] Lee T.H., Chen J.J., Wu C.Y., Yang C.W., Yang H.Y. (2021). Hyperuricemia and Progression of Chronic Kidney Disease: A Review from Physiology and Pathogenesis to the Role of Urate-Lowering Therapy. Diagnostics.

[23] Ma C., Jiang N., Sun X., Kong L., Liang T., Wei X., Wang P. (2023). Progress in optical sensors-based uric acid detection. Biosens. Bioelectron..

[24] Medes G. (1936). Observations on the use of the phosphotungstic acid method of determining ascorbic acid in urines with low ascorbic acid content. Biochem. J..

[25] Yu C.-X., Xiong F., Liu L.-L. (2020). Electrochemical Biosensors with Silver Nanoparticles as Signal Labels. Int. J. Electrochem. Sci..

[26] Sun Y., Saito K., Iiji R., Saito Y. (2019). Application of Ion Chromatography Coupled with Mass Spectrometry for Human Serum and Urine Metabolomics. SLAS Discov..

[27] Moreira O.B.O., Souza J.C.Q., Candido J.M.B., Nascimento M.P., Penna E.A., Chellini P.R., Oliveira M.A.L. (2023). Capillary Electrophoresis Applied to Human Urine Analysis for Clinical Diagnosis: New Trends and Perspectives. Braz. J. Anal. Chem..

[28] Nath P., Mahtab K.R., Ray A. (2023). Fluorescence-Based Portable Assays for Detection of Biological and Chemical Analytes. Sensors.

[29] Malik S., Singh J., Saini K., Chaudhary V., Umar A., Ibrahim A.A., Akbar S., Baskoutas S. (2024). Paper-based sensors: Affordable, versatile, and emerging analyte detection platforms. Anal. Methods.

[30] Chen J., Wang X.L., Wang Y.Q., Zhang Y.F., Peng Z.J., Tang X.M., Hu Y., Qiu P. (2023). Colorimetric detection of uric acid based on enhanced catalytic activity of cobalt-copper bimetallic-modified molybdenum disulfide. Microchem. J..

[31] Lu W.J., Guo Y.J., Yue Y.F., Zhang J.H., Fan L., Li F., Zhao Y., Dong C., Shuang S.M. (2023). Smartphone-assisted colorimetric sensing platform based on molybdenum-doped carbon dots nanozyme for visual monitoring of ampicillin. Chem. Eng. J..

[32] Al-kadumi A.S.H., Abdulsattar J.O., Nabhan K.J. (2020). Colorimetric Determination of Uric Acid in Live samples. Indian J. Forensic Med. Toxicol..

[33] Wu Y., Feng J., Hu G., Zhang E., Yu H.-H. (2023). Colorimetric Sensors for Chemical and Biological Sensing Applications. Sensors.

[34] Lee Y., Haizan I., Sim S.B., Choi J.-H. (2025). Colorimetric Biosensors: Advancements in Nanomaterials and Cutting-Edge Detection Strategies. Biosensors.

[35] Sun L., Shen H., Zheng L., Gao P., Xiang Z. (2021). Colorimetric Detection of Uric Acid Based on Peroxidase-Like Activity of Ag_2_V_4_O_11_ Nanobelts. J. Electron. Mater..

[36] Alberti G., Zanoni C., Magnaghi L.R., Biesuz R. (2021). Gold and Silver Nanoparticle-Based Colorimetric Sensors: New Trends and Applications. Chemosensors.

[37] Mughal S.S., Abbas F., Tahir M.U., Ayub A.R., Javed H.M., Mamtaz M., Iram H. (2019). Role of Silver Nanoparticles in Colorimetric Detection of Biomolecules. Biomed. Nurs..

[38] He S., Lin X., Liang H., Xiao F., Li F., Liu C., Fan P., Yang S., Liu Y. (2019). Colorimetric detection of Cr(VI) using silver nanoparticles functionalized with PVP. Anal. Methods.

[39] Durmazel S., Üzer A., Erbil B., Sayın B., Apak R. (2019). Silver Nanoparticle Formation-Based Colorimetric Determination of Reducing Sugars in Food Extracts via Tollens’ Reagent. ACS Omega.

[40] Bhatt S., Vyas G., Paul P. (2022). Rosmarinic Acid-Capped Silver Nanoparticles for Colorimetric Detection of CN^–^ and Redox-Modulated Surface Reaction-Aided Detection of Cr(VI) in Water. ACS Omega.

[41] Aijaz A., Raja D.A., Khan F.-A., Barek J., Malik M.I. (2023). A Silver Nanoparticles-Based Selective and Sensitive Colorimetric Assay for Ciprofloxacin in Biological, Environmental, and Commercial Samples. Chemosensors.

[42] Uzunoğlu D., Ergüt M., Kodaman C.G., Özer A. (2024). Biosynthesized Silver Nanoparticles for Colorimetric Detection of Fe^3+^ Ions. Arab. J. Sci. Eng..

[43] Singh R., Mehra R., Walia A., Gupta S., Chawla P., Kumar H., Thakur A., Kaushik R., Kumar N. (2023). Colorimetric sensing approaches based on silver nanoparticles aggregation for determination of toxic metal ions in water sample: A review. Int. J. Environ. Anal. Chem..

[44] Rolim W.R., Pelegrino M.T., Lima B.A., Ferraz L.S., Costa F.N., Bernardes J.S., Rodigues T., Brocchi M., Seabra A.B. (2019). Green tea extract mediated biogenic synthesis of silver nanoparticles: Characterization, cytotoxicity evaluation and antibacterial activity. Appl. Surf. Sci..

[45] Verma A., Mehata M.S. (2016). Controllable synthesis of silver nanoparticles using Neem leaves and their antimicrobial activity. J. Radiat. Res. Appl. Sci..

[46] Logaranjan K., Raiza A.J., Gopinath S.C.B., Chen Y., Pandian K. (2016). Shape- and Size-Controlled Synthesis of Silver Nanoparticles Using *Aloe vera* Plant Extract and Their Antimicrobial Activity. Nanoscale Res. Lett..

[47] Jara N., Milán N.S., Rahman A., Mouheb L., Boffito D.C., Jeffryes C., Dahoumane S.A. (2021). Photochemical Synthesis of Gold and Silver Nanoparticles—A review. Molecules.

[48] Ramesh P.S., Kokila T., Geetha D. (2015). Plant mediated green synthesis and antibacterial activity of silver nanoparticles using *Emblica officinalis* fruit extract. Spectrochim. Acta A Mol. Biomol. Spectrosc..

[49] Chutrakulwong F., Thamaphat K., Intarasawang M. (2024). Investigating UV-Irradiation Parameters in the Green Synthesis of Silver Nanoparticles from Water Hyacinth Leaf Extract: Optimization for Future Sensor Applications. Nanomaterials.

[50] Brauen S., Erpf P., Wasem M. (2020). On Absolute and Relative Change. SSRN Electron. J..

[51] Uhrovčík J. (2014). Strategy for determination of LOD and LOQ values—Some basic aspects. Talanta.

[52] Evanoff D.D., Chumanov G. (2005). Synthesis and optical properties of silver nanoparticles and arrays. ChemPhysChem.

[53] Wu D., Lu H.-F., Xie H., Wu J., Wang C.-M., Zhang Q.-L. (2015). Uricase-stimulated etching of silver nanoprisms for highly selective and sensitive colorimetric detection of uric acid in human serum. Sens. Actuators B Chem..

[54] Parnklang T., Lertvachirapaiboon C., Pienpinijtham P., Wongravee K., Thammacharoen C., Ekgasit S. (2013). H_2_O_2_-triggered shape transformation of silver nanospheres to nanoprisms with controllable longitudinal LSPR wavelengths. RSC Adv..

[55] He D., Garg S., Waite T.D. (2012). H_2_O_2_-Mediated Oxidation of Zero-Valent Silver and Resultant Interactions among Silver Nanoparticles, Silver Ions, and Reactive Oxygen Species. Langmuir.

[56] Filippo E., Serra A., Manno D. (2009). Poly(vinyl alcohol) capped silver nanoparticles as localized surface plasmon resonance-based hydrogen peroxide sensor. Sens. Actuators B Chem..

[57] Nishan U., Ahmed A., Muhammad N., Shah M., Asad M., Khan N., Ullah F., Ullah R., Ali E.A., Nawaz H. (2024). Uric acid quantification via colorimetric detection utilizing silver oxide-modified activated carbon nanoparticles functionalized with ionic liquid. RSC Adv..

[58] Li L., Wang J., Chen Z. (2020). Colorimetric determination of uric acid based on the suppression of oxidative etching of silver nanoparticles by chloroauric acid. Microchim. Acta.

[59] Nayak S.P., Ramamurthy S.S., Kiran Kumar J.K. (2020). Green synthesis of silver nanoparticles decorated reduced graphene oxide nanocomposite as an electrocatalytic platform for the simultaneous detection of dopamine and uric acid. Mater. Chem. Phys..

[60] Amjadi M., Hallaj T., Nasirloo E. (2020). In situ formation of Ag/Au nanorods as a platform to design a non-aggregation colorimetric assay for uric acid detection in biological fluids. Microchem. J..

[61] Yang X., Long S., Wang B., Chen J., Xiong Y., Ying M. (2025). Biohybrid Nanosystem Fabricated with Marine Diatom *Thalassiosira pseudonana* for Uric Acid Detection. ACS Biomater. Sci. Eng..

[62] Nishan U., Khan N., Muhammad N., Afridi S., Badshah A., Shah M., Asad M., Ullah R., Niamat H., Ullah R. (2023). Colorimetric sensing of uric acid based on sawdust-deposited silver nanoparticles via an eco-friendly and cost-effective approach. Front. Mater..

[63] Zhang Q., Du S., Tian F., Long X., Xie S., Tang S., Bao L. (2022). Silver Nanoparticle-Functionalised Nitrogen-Doped Carbon Quantum Dots for the Highly Efficient Determination of Uric Acid. Molecules.

[64] Wang X., Lu J., Tang X., Qiu P. (2020). Colorimetric Detection of Uric Acid with High Sensitivity Using Cu_2_O@Ag Nanocomposites. Chem. Afr..

